# Electrochemical route to the synthesis of ZnO microstructures: its nestlike structure and holding of Ag particles

**DOI:** 10.1186/1556-276X-8-78

**Published:** 2013-02-15

**Authors:** Ling Ding, Ruixue Zhang, Louzhen Fan

**Affiliations:** 1 Department of Chemistry, Beijing Normal University, 100875, Beijing, China

**Keywords:** ZnO microstructures, Nestlike structure, Ag-ZnO nestlike heterostructure, Electrodeposition

## Abstract

**Abstract:**

A simple and facile electrochemical route was developed for the shape-selective synthesis of large-scaled series of ZnO microstructures, including petal, flower, sphere, nest and clew aggregates of ZnO laminas at room temperature. This route is based on sodium citrate-directed crystallization. In the system, sodium citrate can greatly promote ZnO to nucleate and directly grow by selectively capping the specific ZnO facets because of its excellent adsorption ability. The morphology of ZnO is tuned by readily adjusting the concentration of sodium citrate and the electrodeposition time. Among the series structures, the remarkable ZnO nestlike structure can be used as a container to hold not only the interlaced ZnO laminas but also Ag nanoparticles in the center. The special heterostructures of nestlike ZnO holding Ag nanoparticles were found to display the superior properties on the surface-enhanced Raman scattering. This work has signified an important methodology to produce a wide assortment of desired microstructures of ZnO.

**PACS:**

81 Materials science 81.07.-b nanoscale materials and structures Fabrication Characterization 81.15.-z Methods of deposition of films Coatings Film growth and epitaxy.

## Background

Construction of micro- and nanoscale semiconductor materials with special size, morphology, and hierarchy has attracted considerable attention for potential application due to their distinctive functions, novel properties, and potential applications in advanced devices and biotechnologies [1,2]. Rational control over the experimental condition has become a hot topic in recent material research fields. ZnO is currently one of the most attractive semiconducting materials for optical and electronic applications because of its direct wide band gap (3.37 eV) and high exciton binding energy (60 meV) [3]. Since Yang observed the room temperature UV lasing from ZnO nanorod arrays [4], much effort has been devoted to tailor the morphology and size to optimize the optical properties. As a result, various ZnO nanostructures, including nanowires [5-7], nanotubes [8,9], nanobelts [10], nanoflowers [11], nanospheres [12], nanobowls [13], dandelions [14], cages [15], and shells [16,17] have been obtained by solid-vapor phase growth [18], microemulation [19], and hydrothermal methods [20,21]. Hereunto, nanobowls, nanocups, or nanodishes have attracted much interest because they have been envisaged to further contain nanoparticles [22] and immobilize biomolecules [23,24]. Although conventional methods can produce various ZnO micro-/nanostructures, these different synthesis methods often greatly suffer from problems of high temperature, need for high vacuum, lack of control, and high cost. To develop a simple, fast, and controllable synthetic route that can not only control the ZnO micro-/nanostructures with series of shapes under ambient condition, but also produce the hierarchical structure, remains an important topic of investigation.

The electrochemical deposition technique has been recently developed as a promising alternative means for the fabrication of nanomaterials under ambient condition due to the low cost, mild condition, and accurate process control. Recently, Yang and co-workers [25] reported the synthesis of ultrathin ZnO nanorods/nanobelts arrays on Zn substrates by electrochemical deposition. Our group [26] reported an electrochemical route for the fabrication of highly dispersed composites of ZnO/carbon nanotubes. Herein, we report a tunable self-assemble strategy to selectively fabricate a series of ZnO with unique, pure, and larger quantity morphologies including petal-, flower-, sphere-, nest- and clew-shaped structures by electrochemical deposition. The size and morphology of the ZnO are systematically controlled by judiciously adjusting the concentration of the sodium citrate and the electrodepositing time in the self-assembly process. Significantly, the nestlike structure dominates the further formation of hierarchical superstructure. The ZnO nestlike structure can be used as a container not only to hold several interlaced ZnO laminas, but also to fabricate Ag-ZnO heterostructures by growing silver nanoparticles or clusters in the center of nests by electrochemical deposition method. The multiphonon Raman scattering of as-fabricated Ag-ZnO nestlike heterostructures is also largely enhanced by the strongly localized electromagnetic field of the Ag surface plasmon.

## Methods

### Synthesis of ZnO microstructures

Zinc foils (99.9%, Sigma-Aldrich Corporation, St. Louis, MO, USA) with a thickness of 0.25 mm were polished by sand paper then ultrasonically washed in absolute ethanol and dried in air before use. Electrochemical experiments with a CHI workstation were performed at room temperature in a two-electrode (Zn-Zn) system. For the production of nestlike ZnO, 0.01 mmol of sodium citrate and 14 μl of 30% H_2_O_2_ were added to 7 ml of deionized water under stirring at room temperature, adjusting the pH to 12. The two Zn foils (5 × 5 × 0.25 mm^3^) were put into the reaction solution in a parallel configuration with an interelectrode separation of 1 cm to apply a fixed electric potential of 3 V between the two Zn electrodes by using the electrochemical analyzer for the electrochemical deposition of ZnO nanostructures at room temperature. After being electrodeposited for 1 min, a whitish gray film was generated on the surface of Zn cathode. The Zn cathode with the deposited products was washed with distilled water for several times, dried at room temperature, and examined in terms of their structural, chemical, and optical properties. The ZnO petals, flowers, clews, and microspheres were fabricated by varying the molar quantity of sodium citrate and the deposition time synchronously, while keeping the other experimental conditions identical.

### Preparation of Ag/ZnO heterostructures

A conventional cell with a three-electrode configuration was used throughout this work. The Zn cathode with the deposited nestlike ZnO structures was employed as the working electrode. A Pt wire served as the counter electrode, and the Ag/AgCl electrode was used as the reference electrode. The working electrode was biased at −0.6 V in 0.001 M AgNO_3_ solution for 1 min. Then the Ag clusters which were conglomerated by Ag nanoparticles were held in the center of ZnO nestlike structures on the surface of Zn cathode.

### Structural characterizations

The as-prepared multiform ZnO microstructures or nanostructures and Ag/ZnO heterostructures on Zn foils were directly subjected to characterizations by the Hitachi S4800 scanning electron microscope (SEM; Hitachi High-Technologies Corporation, Tokyo, Japan) and the JEOL 2010F transmission electron microscope (TEM; JEOL Ltd., Tokyo, Japan) with high-resolution TEM imaging and energy dispersive X-ray. The samples used for TEM measurement were prepared by dispersing some products scraped from the Zn cathode in ethanol, then placing a drop of the solution onto a copper grid and letting the ethanol evaporate slowly in air. X-ray powder diffraction (XRD) measurement was performed on a Shimadzu XRD-6000 (Shimadzu Co. Ltd., Beijing, China) using Cu Kα radiation (1.5406 À) of 40 kV and 20 mA. Photoluminescence spectra were measured at room temperature using a Xe laser as an excitation source with a LS50 steady-state fluorescence spectrometer (Shimadzu, RF-5301PC). The resonant Raman spectra were performed using a Jobin Yvon LabRAM HR 800 UV micro-Raman spectrophotometer (Horiba Instruments, Kyoto, Japan) at room temperature. The 325-nm line of the He-Ne laser served as excitation light source.

## Results and discussion

**Figure 1:**
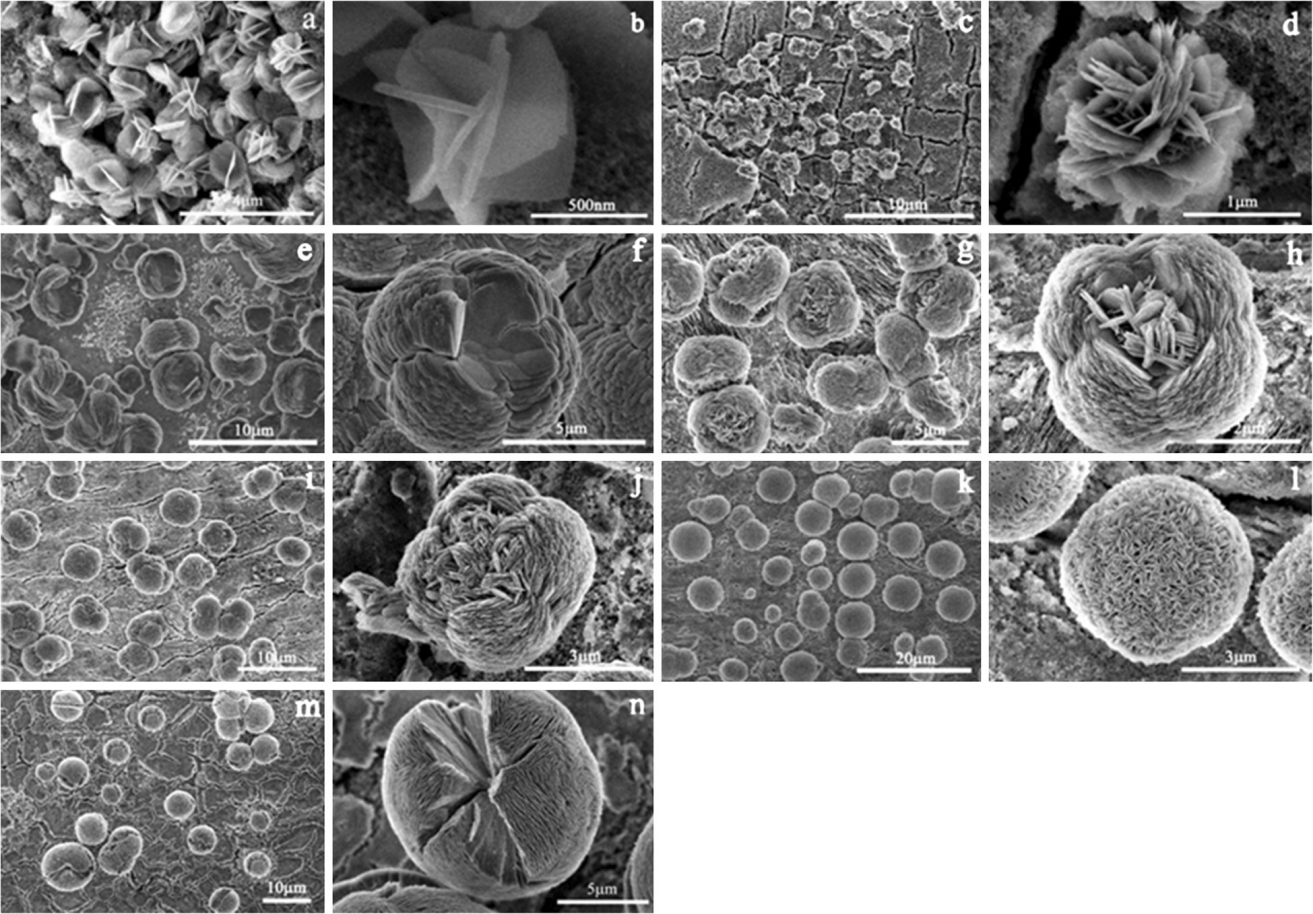
**SEM images of different ZnO microstructures by varying the electrochemical deposition conditions.** (**a**, **b**) 0.05 mmol, 1 min; (**c**, **d**) 0.1 mmol, 3 min; (**e**, **f**) 0.01 mmol, 3 min; (**g**, **h**) 0.05 mmol, 5 min; (**i**, **j**) 0.05 mmol, 30 min; (**k**, **l**) 0.1 mmol, 5 min; (**m**, **n**) 0.1 mmol, 10 min.

Different ZnO morphologies can be selectively obtained by simply varying the concentration of sodium citrate and the electrodeposition time within the certain pH range and supplying current (shown in Figure 1). The image of the small petals intersected by some laminas in one another is shown in Figure 1a,b by controlling the concentration of sodium citrate of 0.05 mmol for deposition time of 1 min at room temperature. The average size of these small petals is about 800 nm. In 0.1 mmol of sodium citrate at deposition time of 3 min, the compact ZnO flowers with average diameter of 1 to 2 μm are formed (Figure 1c,d). The microstructure is actually composed of a random growth of seemingly flexible nanolaminas that can be bent and connected with each other. The nanolaminas extend from the center of the microflowers outward. The ZnO nestlike structures with concave centers are obtained in good yield with a diameter from 2 to 5 μm (Figure 1e,f) for the electrochemical deposition of 1 min in the presence of 0.01 mmol sodium citrate aqueous solution. The shape of the hatch of these nests is square, and the length of the sides is about 4 μm. Figure 1f shows that the nestlike structure is composed of densely packed layers from the bottom to the top. Every layer consists of four well-edged square nanolaminas with the side length of about 2 μm. At the base of the nestlike structure in Figure 1e, if the concentration of sodium citrate is changed to 0.05 mmol with the deposition time of 5 min, ZnO nests holding the interlaced nanolaminas of ZnO are obtained (Figure 1g,h). The ZnO nanolaminas located in the center of ZnO nests are analogy to the flower pistil. Many of these flower pistils show secondary laminas, which have started to grow on the concave of the nests with a slightly different orientation: the secondary laminas form an angle with the basal plane of the main structure and trend to self-assemble in the center of the nests. With the electrochemical deposition going on, the central cavity of the nest is gradually filled by the nanolaminas to form clew-like structure (Figure 1i,j). However, the different growth directions for the nest and its pistil are easily recognized from their gap (Figure 1j). Using 0.1 mmol sodium citrate at deposition time of 5 min, the flower-like microstructure of Figure 1d gradually disappeared and transformed into microsphere structure with an average diameter of 5 μm (Figure 1k,l). These ZnO microspheres are in fact built from small one-dimensional nanolaminas in a highly close-packed assembly. These nanolaminas are aligned with one another perpendicularly to the more compact ZnO spherical surface. The nanolaminas also served as new nucleation sites for more nanolaminas growth and the eventual development into a well-defined three-dimensional spherical structure. But when further increasing the reaction time to 10 min and keeping the concentration of sodium citrate certain, nearly all of the ZnO microspheres show large cracks along the equatorial circumference in Figure 1m,n, which may be due to the slightly increased tension of the inner spheres.

**Figure 2:**
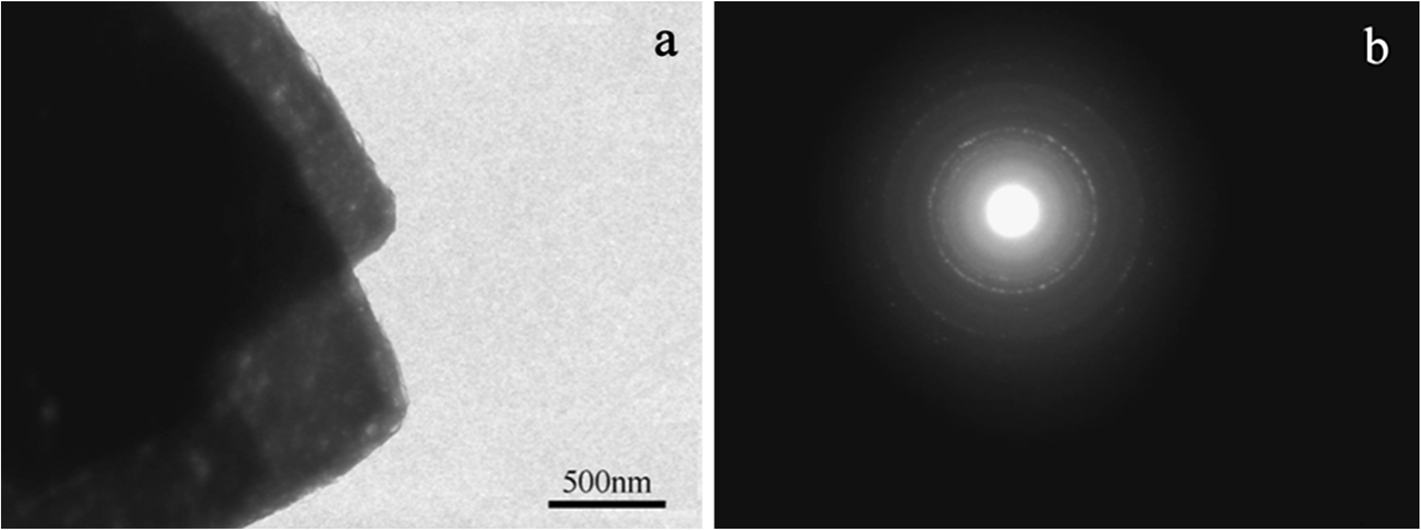
TEM image (a) and ED ring of laminas of ZnO structures (b).

The TEM image of the two typical broken laminas of ZnO from any structure in Figure 1 obtained by ultrasonic treatment for several minutes is shown in Figure 2a. The electron diffraction (ED) pattern (Figure 2b) of these nanolaminas suggests that they have a polycrystalline structure [8].

A serials of experiments showed that the existence of citrate ions played a key role in the formation of the ZnO complex microstructures. For the control experiment in the absence of citrate as we previously reported, the products were mainly nanoflowers which were composed of nanorods [26]. When citrate was introduced in the solution, the period for the formation of ZnO nuclei (induction and latent periods in the crystal growth process) [27] was remarkably prolonged, which suggested that citrate could slow down the nucleation and subsequent crystal growth of ZnO. On the basis of the previous analysis, we proposed a reasonable mechanism for the formation of ZnO structures. It is believed that sodium citrate is extensively used as the stabilizer and structure-directing agent because of its excellent adsorption ability [28,29]. The additive citrate can form strong complexes [Zn(C_6_H_5_O_7_)_4_]^10−^ with Zn^2+^ and owing to the stability of [Zn(C_6_H_5_O_7_)_4_]^10−^ which is larger than [Zn(OH)_4_]^2−^ in the present situation, there exists a large quantity of [Zn(C_6_H_5_O_7_)_4_]^10−^ with negative charge and a small quantity of [Zn(OH) _4_]^2−^ in the precursor solution. It has been previously reported that citrate anions have been known to act as a capping agent of the (0001) surface of the ZnO crystal by adsorbing on the positive polar face of the (0001) surface [30,31]. Thus, these [Zn(C_6_H_5_O_7_)_4_]^10−^ ions are preferred to absorb positive polar plane (0001) surface through the -COO^−^ and -OH functions, and decrease the growth rate of (0001) ZnO crystal surface by competing with growth units [Zn(OH)_4_]^2−^, which limits the anisotropy growth of ZnO at experimental pH value and leads to the formation of lamina-like ZnO nanostructures, as shown in Figure 1a,b. The stacking of the laminas is not completely ordered, and the laminas’ self-assembly at a later time is progressively more tilted leading to the formation of petal-like, flower-like, nestlike, clew-like, and spherical aggregates for adjusting the electrodeposition time and the concentration of sodium citrate.

It is worth mentioning that the morphologies of the products varied remarkably with the concentration of citrate. On the basis of the experiment results, we found that when the concentration of citrate was lower than 0.05 mmol (0.01 mmol in Figure 1e,f), the nascent square nanolaminas would self-assemble from bottom to top to form nestlike structures. On the other way around, when the concentration of citrate was higher than 0.05 mmol (0.1 mmol in Figure 1d,l,n), the nascent nanolaminas would self-assemble from center outwards to generate flower-like or microsphere structures. It has been reported that high citrate concentration (higher than 0.05 mmol) will attain [Zn(C_6_H_5_O_7_)_4_]^10−^ supersaturated solution and Ostwald ripening controls structure growth by the diffusion of [Zn(C_6_H_5_O_7_)_4_]^10−^ ions along the matrix-particle boundary tending to form spherical/hemispherical shapes from the center [32,33]. In contrary to this, the lower citrate concentrations will not form [Zn(C_6_H_5_O_7_)_4_]^10−^ supersaturated solution, which tend to self-assemble from bottom to top.

**Figure 3:**
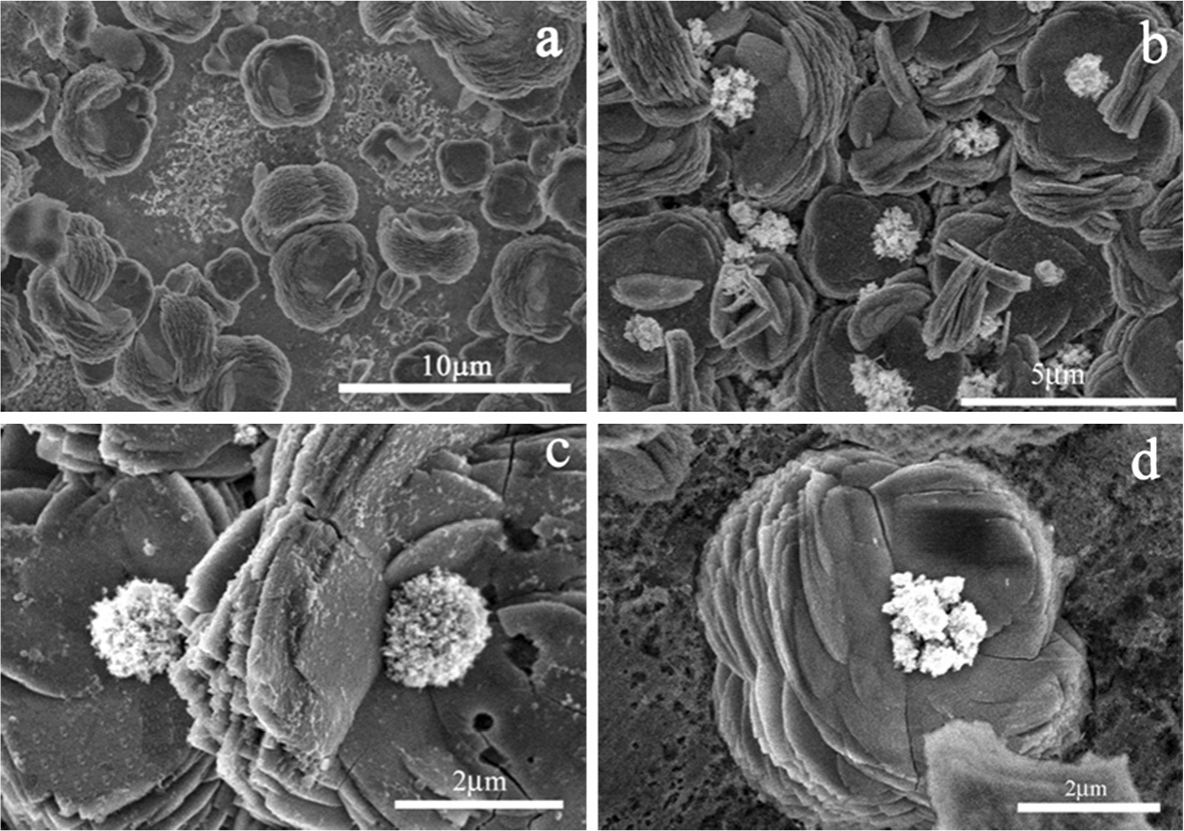
SEM images of blank ZnO nestlike structures (a) and Ag-ZnO nestlike heterostructures (b,c,d).

Through the above morphological controls, we have succeeded in preparing a series of ZnO with unique morphologies including petal-, flower-, sphere-, nest- and clew-shaped structures by electrochemical deposition self-assembly. The distinctive nestlike ZnO structures have provided opportunities for creating more sophisticated structures. Figure 1h,g has clearly demonstrated that it can hold ZnO laminas as a pistil. Then we further place silver nanoparticles or nanoclusters in the center of ZnO nests by electrochemical deposition. Figure 3a shows the SEM image of blank ZnO nests. Figure 3b,c,d show the typical results of the ZnO nests after the silver deposition at −0.6 V for 1 min. It can be clearly seen that the nanosized silver particles or silver clusters are apt to form in the center of each ZnO nests. Nearly no silver clusters structures or particles were found outside of the nestlike structures. This indicates that the formation of the silver nanostructures exhibits a location-selective property. Namely, the center of ZnO nests is the place where the Ag nanostructures formed facilely, likely because it is close to the surface of the electrode.

**Figure 4:**
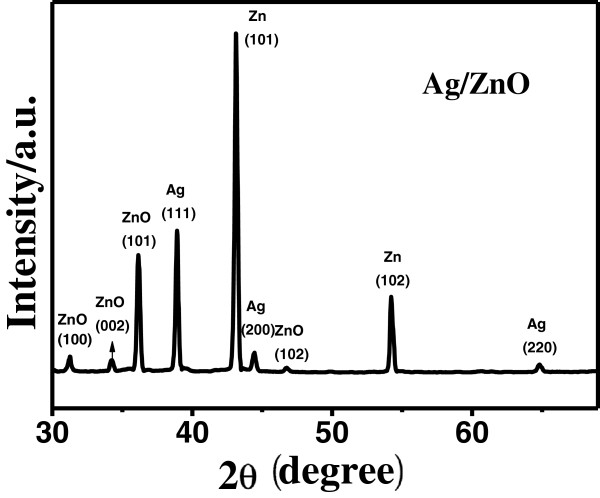
XRD patterns of Ag-ZnO nestlike heterostructures.

The XRD pattern of Ag-ZnO nestlike heterostructures is shown in Figure 4. The Zn(101) and (102) peaks can be observed due to the used Zn foil substrate (JCPDS card number 040831). These (100), (002), (101), and (102) peaks can be indexed to hexagonal wurtzite ZnO (JCPDS card number 361451). The appearance of the Ag(111), (200), and (220) peaks provides evidence that crystalline Ag is formed in the nestlike ZnO, with the (111) peak being especially strong. The three reflection peaks can be indexed to the Ag face-centered cubic crystal structure compared with the standard JCPDS card (040783). In addition no diffraction peaks from the other crystalline forms are detected.

**Figure 5:**
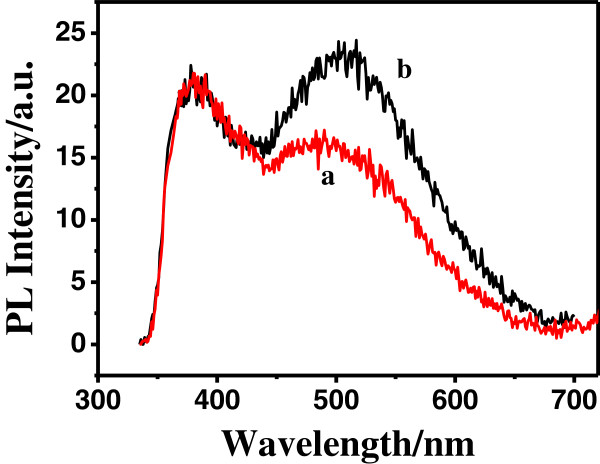
PL emission spectra (λ ex = 325 nm) of the Ag/ZnO heterostructures (a) and blank ZnO nestlike structures (b).

The photoluminescence (PL) spectra of the as-synthesized Ag-ZnO nestlike heterostructures together with blank nestlike ZnO as a comparison were investigated. As shown in Figure 5, a broad green emission peak centering at around 505 nm is observed in the visible region when the samples are excited at 325 nm. Despite the intensive studies on the green emission of ZnO crystals, its nature remains controversial, and a number of hypotheses have been proposed to explain this emission, such as a singly ionized oxygen vacancy [34], an oxygen antisite defect [35], and a zinc vacancy [36]. We ascribe the green emission at about 505 nm to the singly ionized oxygen vacancy on the surface of ZnO structures. It is obvious that the green emission intensity of the as-synthesized Ag-ZnO nestlike heterostructures decreases when compared with the blank nestlike ZnO. This phenomenon reveals that the decrease of the ionized oxygen defect density on the surface of ZnO nests in the Ag-ZnO nestlike heterostructures is due to the holding Ag nanoparticles in the center of the nestlike ZnO. In comparison with the blank nestlike ZnO nanostructures, the Ag-ZnO nestlike heterostructures show no shift in the UV region, and a large (approximately 20 nm) blue shift in the visible emission which could be attributed to the interaction between Ag and ZnO resulting in the Fermi level of ZnO is changed. The quenching of the trapped emission is expected via the new nonradiative pathways created by the proximity of the metal, possibly resulting from electron transfer from ZnO to Ag [37].

**Figure 6:**
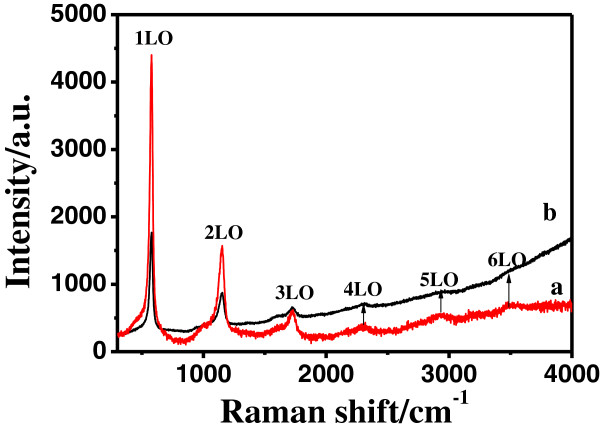
**Enhanced Raman scattering of Ag-ZnO nestlike heterostructures.** (**a**) relative to blank ZnO nestlike structures (**b**) using a He-Ne laser (λ = 325 nm).

In order to further detect the interface between ZnO and Ag, surface-enhanced Raman scattering (SERS) spectrum was measured for Ag-ZnO nestlike heterostructures with blank nestlike ZnO as comparison (Figure 6). As is evident from the curve b, blank nestlike ZnO has weaker Raman signal. However, for the Ag-ZnO nestlike heterostructures (curve a), a strong Raman scattering line is observed at 578, 1,153, and 1,726 cm^−1^ which is assigned to the ZnO 1LO, 2LO, and 3LO modes [38]. The 1LO photo mode of the Ag-ZnO nestlike heterostructures shows threefold enhancement compared to that of blank nestlike ZnO. In addition the 4LO (2,318 cm^−1^), 5LO (2,932 cm^−1^), and 6LO (3,506 cm^−1^) [39] can be observed distinctly when Ag nanoparticles were deposited in the center of ZnO nests. In the range of larger wavelength, the baseline of the Raman intensity has declined. This phenomenon might be associated with the quenching fluorescence of ZnO in the Ag-ZnO nestlike heterostructures. Theoretical and experimental studies on SERS mechanisms have revealed that the SERS signals are primarily attributed to the electromagnetic excitation of strongly localized surface plasmon of noble metals [40]. In the Ag-ZnO nestlike heterostructures, we also count the localized electromagnetic effect of the Ag surface plasmon as mostly responsible for the enhancement of multiphonon Raman scattering. In addition, based on the fact that surface plasmon energy of metal Ag matches well with the emitted visible photon energy from the ZnO, the surface plasmon of the Ag nanoparticles might be resonantly excited through energy transfer in the near field and create a stronger local electromagnetic field [41]. The incident light field coupling to the local surface plasmon field might induce stronger localized electromagnetic field in the interface between ZnO and Ag, which further enhances the multiphonon Raman scattering of ZnO, demonstrating the formation of Ag-ZnO heterostructures.

## Conclusions

In summary, a convenient approach based on sodium citrate as capping reagent has been developed for the shape-selective synthesis of ZnO with controllable morphologies at room temperature by electrochemical deposition. Four important results were highlighted in this work:

(1) The room-temperature synthesis of ZnO with controllable morphologies, such as petal-, flower-, sphere-, nest-, and clew-shaped structures with adjusting the concentration of sodium citrate, and the deposition time by electrochemical route, has been realized for the first time. Only one or a few kinds of shapes within a narrow size range can be achieved from one of the previous methods [42]. This result should facilitate the development of an effective and low-cost fabrication process for high-quality ZnO.

(2) The product morphologies and sizes were highly controllable and modifiable and evolved from several micro-compressed laminas to flowerlike structures assembled by laminas and to the nestlike microstructure and microsphere in last.

(3) The nest-shaped ZnO microstructures consisting of nanolaminas have been successfully synthesized by using sodium citrate. Our experimental results indicate that the ZnO nestlike structures can be used as a container not only to hold lamina-like ZnO, but also to be used to grow silver nanoparticles in the center of ZnO nests by electrochemical method.

(4) The optical properties (PL and SERS) of the ZnO nests holding nanoparticles of Ag exhibit strong coupling between the metal and semiconductor.

## Abbreviations

ED: electron diffraction;PL: photoluminescence;SEM: scanning electron microscope;SERS: surface-enhanced Raman scattering;TEM: transmission electron microscope;XRD: X-ray powder diffraction

## Competing interests

The authors declare that they have no competing interests.

## Authors’ contributions

LD performed the experiment and drafted the manuscript, RZ proposed the idea and participated in the experiment. LF supervised the work and finalized the manuscript. All authors read and approved the final manuscript.
